# In Memoriam

**Published:** 1876-03

**Authors:** Thos. D. Strong, C. H. Richmond, H. D. Vosburgh

**Affiliations:** Committee of Curators; Committee of Curators; Committee of Curators


### In Memoriam.

At a meeting of the Board of Curators of the Medical Department of the University of Buffalo, held at the College Monday evening, Feb. 21, the following resolutions were prepared by the Committee named, and adopted as the sense of the Board:

Whereas, In the Providence of God our esteemed friend and fellow-citizen, Dr. Milton E. Potter, of Attica, N. Y., in the fulness of years and after a useful and laborious life, has passed away since our last meeting; and

Whereas, In his death we lose not only a worthy and honored member of our profession, but also a tried and trusty friend of the Buffalo Medical College, an active Curator, and a cultivated man; therefore

*Resolved*, That in his death we recognize the hand of a wise, overruling Providence governing the lives and affairs of men in ways inscrutable to us.

*Resolved*, That in Dr. Milton E. Potter we recognized a representative physician, a man full of labors and good works, whose busy life has been so fully spent for others in the labors of his profession as to merit richly the words, "Well done, thou good and faithful servant."

*Resolved*, That in the death of Dr. Potter we lose one of our most esteemed fellows, and the Buffalo Medical College one of its most active and trusted friends.

*Resolved*, That a copy of these resoulutions be presented for publication to the Buffalo papers, the Buffalo MEDICAL AND SURGICAL JOURNAL, and a copy duly signed be sent to the family in token of our appreciation of the deceased.

THOS. D. STRONG, M. D.,
C. H. RICHMOND, M. D.,
H. D. VOSBURGH, M. D.,
Committee of Curators.

